# Deep-Learning-Driven Full-Waveform Inversion for Ultrasound Breast Imaging

**DOI:** 10.3390/s21134570

**Published:** 2021-07-03

**Authors:** Thomas Robins, Jorge Camacho, Oscar Calderon Agudo, Joaquin L. Herraiz, Lluís Guasch

**Affiliations:** 1 Department of Earth Science and Engineering, Faculty of Engineering, Imperial College London, London SW7 2AZ, UK; thomas.robins11@imperial.ac.uk (T.R.); o.calderon-agudo14@imperial.ac.uk (O.C.A.); 2 Ultrasound Systems and Technology Group (GSTU), Institute for Physical and Information Technologies (ITEFI), Spanish National Research Council (CSIC), 28006 Madrid, Spain; j.camacho@csic.es; 3 Nuclear Physics Group and IPARCOS, Faculty of Physical Sciences, University Complutense of Madrid, CEI Moncloa, 28040 Madrid, Spain; jlopezhe@ucm.es; 4 Health Research Institute of the Hospital Clínico San Carlos (IdISSC), 28040 Madrid, Spain

**Keywords:** full-waveform inversion, breast imaging, ultrasound tomography, deep learning

## Abstract

Ultrasound breast imaging is a promising alternative to conventional mammography because it does not expose women to harmful ionising radiation and it can successfully image dense breast tissue. However, conventional ultrasound imaging only provides morphological information with limited diagnostic value. Ultrasound computed tomography (USCT) uses energy in both transmission and reflection when imaging the breast to provide more diagnostically relevant quantitative tissue properties, but it is often based on time-of-flight tomography or similar ray approximations of the wave equation, resulting in reconstructed images with low resolution. Full-waveform inversion (FWI) is based on a more accurate approximation of wave-propagation phenomena and can consequently produce very high resolution images using frequencies below 1 megahertz. These low frequencies, however, are not available in most USCT acquisition systems, as they use transducers with central frequencies well above those required in FWI. To circumvent this problem, we designed, trained, and implemented a two-dimensional convolutional neural network to artificially generate missing low frequencies in USCT data. Our results show that FWI reconstructions using experiment data after the application of the proposed method successfully converged, showing good agreement with X-ray CT and reflection ultrasound-tomography images.

## 1. Introduction

Breast cancer is globally the most frequently diagnosed cancer in women, and the most frequent cause of death due to cancer [1]. In recent decades, the introduction of screening programmes to improve early detection and diagnosis of breast cancer has lead to a reduction in the rates of advanced breast cancer and mortality rates [2,3]. Typically, breast-cancer screenings are performed using X-ray mammography due to their high resolution, short acquisition time, and low cost. However, as this modality uses ionising radiation, it poses a non-negligible risk of inducing breast cancer [4]. Additionally, it is not recommended for pregnant women.

Furthermore, despite women with dense breast tissue being more likely to be diagnosed with breast cancer than those with less dense breasts, the sensitivity of mammograms decreases with breast-tissue density, as lesions are more likely to be obscured [5,6]. This has led to a significantly high rate of false negatives in younger women and women with dense breast tissue [7].

In cases where it is not possible to distinguish between cancerous and dense breast tissue, high-frequency US is used to complement mammograms and better characterise lesions [8]. In addition to being better suited for imaging dense breast tissue, ultrasound-imaging devices are less expensive than the hardware and infrastructure costs required to safely perform X-ray mammography. Furthermore, as ionising radiation is not required, this modality is also significantly safer. However, the conventional pulse-echo ultrasound imaging of the whole breast is currently a time-consuming procedure that needs to be carried out by specialized physicians using hand-held probes, making this approach more costly to perform compared to mammography. Evaluating lesions using ultrasound breast imaging is also highly dependent on the expertise of the clinician, and does not generate high-quality images that can be assessed at a later date. For these reasons, conventional pulse-echo ultrasound is not considered to be suitable as the sole imaging modality for screening programs.

To overcome the limitations of conventional ultrasound in breast imaging, ultrasound computed tomography (USCT) was proposed as a safer and more affordable alternative to mammography. With this approach, hand-held imaging is replaced with arrays of ultrasound transducers that completely surround imaging targets, allowing for the recording of both reflection and transmission data. With these data, tomographic algorithms can be used to reconstruct quantitative models of acoustic breast-tissue properties such as speed of sound (SoS), density, and sound attenuation, allowing for the classification of different breast tissue types and the detection of cancerous masses [9].

In conventional USCT techniques, such as time-of-flight (TOF) tomography, ray approximation is used to model the wave propagation between source and receiver elements of the transducer array. Here, delays related to changes in tissue SoS along the trajectory of these ray paths are found by estimating the first arrival times between source–receiver pairs, and can be used to reconstruct speed-of-sound models of the breast. This approximation provides a computationally inexpensive method for producing breast tomograms [10]. This allows for clinicians to analyse the resulting quantitative models at a later date using diagnostic imaging software, making this procedure more cost-effective than conventional ultrasound breast imaging is. However, the maximal theoretical spatial resolution that can be achieved using this oversimplified model of wave behaviour is limited by the order of the diameter of the first Fresnel zone [11]. This prevents this reconstruction approach from being diagnostically useful when screening for breast cancer.

Improvements in available computing power for medical-imaging applications recently allowed for more computationally expensive USCT techniques to be considered for breast imaging, such as full-waveform inversion (FWI), an iterative data-fitting method that was originally developed for seismic exploration. FWI can be used to produce high-quality, high-resolution reconstructions of 3D breast tissue compared to ray-based USCT methods [12,13]. To perform FWI, a forward model with a numerical wave equation is used to simulate wave propagation through an acoustic model of the breast being imaged that, when sampled at receiver positions, provides a corresponding synthetic dataset for all source–receiver pairs observed in real data. This acoustic breast model is then updated at each iteration, such that the misfit between observed and synthetic data is minimised. As the full propagation of the wave is modelled, this approach is able to account for multiple scattering in the wavefield, and resolve features at subwavelength resolutions, significantly improving the spatial resolution that can be achieved using ray-based methods.

Successful reconstructions using conventional FWI are dependent on how close the initial acoustic model is to the true solution and whether the low-frequency content of the signal is sufficiently low enough to mitigate cycle skipping, a well-documented problem in applications of FWI in geophysics. This occurs when the misfit between the lowest-frequency signal content of the observed and synthetic data exceeds a half-cycle difference in time, causing the inversion to converge to the local minima [14]. This makes it challenging to invert band-limited acquisitions with insufficient low-frequency content. As demonstrated by an in silico FWI breast imaging study by Calderon Agudo et al. [12], USCT data with frequencies below 0.50 MHz were required to mitigate cycle skipping when using a conventional FWI algorithm. However, medical ultrasound-imaging probes are not typically designed to image sub-1.00 MHz frequencies, making them poorly suited to this task.

Mathematical low-frequency extrapolation methods were investigated to generate low-frequency signal content that may be missing from acquired data (Li and Demanet et al. [15]). However, these bandwidth extension approaches are recognised as nonlinear operations that add additional costs and are only able to estimate low-frequency content in controlled situations. An alternative approach to using a physics-based bandwidth extension algorithm is to instead pose this task as a machine-learning problem. This concept is supported by the universal-approximation theorem that states that a deep neural network (DNN) with a sufficient number of hidden layers can be used to approximate any nonlinear function [16]. For this application, this DNN would be used to approximate an operation that directly extrapolates the true low-frequency signal phase and amplitude values from band-limited signal data. This was demonstrated for geophysical applications by Sun et al. using a 1D convolutional neural network (CNN) that was trained to perform this task using synthetic data of a signal transmitted through acoustic subsurface models [17].

In this study, we further explore this approach for bandwidth extension by implementing a U-Net-based 2D CNN architecture to preserve the signal structure along the time axis and across transducer channels when extrapolating low-frequency signal content. In Section 2, we describe the architecture of this proposed bandwidth extension CNN, and the methodology for generating realistic in silico USCT breast imaging data for training and evaluating the CNN solutions. Furthermore, we provide an overview of the USCT acquisition systems used to provide experimental USCT data of a realistic breast phantom. In Section 3, we discuss the results from applying the CNN solutions to both in silico and experimental band-limited FWI breast imaging problems. Lastly, in Section 4, we discuss the impact of the proposed bandwidth-extension solution to overcome the inherent limitations of band-limited ultrasound hardware when imaging the breast with FWI.

## 2. Materials and Methods

Here, we discuss the methodology used for training and testing the bandwidth extension CNN solutions for the low-frequency extrapolation of band-limited USCT data. These include PyTorch implementations of both our proposed U-Net based 2D CNN solution and the 1D CNN model described by Sun et al. [17]. Both CNNs were run on a GTX 2080 Ti GPU (Nvidia, Santa Clara, CA, US) using the same training and testing datasets, allowing for the performance of both bandwidth-extension methods to be compared. We then discuss the method used to generate synthetic USCT training, validation, and testing datasets using numerical SoS breast models provided by the OA-Breast database [18]. Lastly, we provide an overview of the experimental acquisition system used to image a CIRS 073 acoustic breast phantom [19] to provide real-world USCT testing data. This system consisted of a pair of translated P4-1 cardiac probes (ATL, Bothell, WA, US) commonly found in clinical settings, and they were selected for being among the lowest-frequency transducers available in medical ultrasound. Despite these selection criteria, we demonstrate that these probes were still unable to image at sufficiently low frequencies to mitigate cycle skipping when using a conventional FWI algorithm without prior information about the breast phantom. Experimental USCT data were, therefore, band-limited, providing an ideal case study for testing our bandwidth-extension CNN solutions. The resulting SoS reconstructions from these tests were validated using a micro-CT scan (using a XT H 225 CT scanner, Nikon, Tokyo, Japan) and ultrasound reflectivity images (performed using a full-angle spatial-compound technique [20] of the CIRS breast phantom.

### 2.1. Bandwidth-Extension Network Architecture

The low-frequency extrapolation method proposed by Sun et al. [17] used a feed-forward CNN model design with an architecture that consisted of five convolutional steps, each with parametric ReLU (PReLU) and batch-normalisation layers, to extrapolate broadband data from 1D trace inputs. This neural network was trained with a supervised-learning approach, where simulated infrasound transmissions (sub-20 Hz) through numerical acoustic subsurface models were used to generate paired band-limited and broadband infrasound acquisitions. These were used as the input and target data, respectively. Once trained, this CNN was sufficiently robust enough to extrapolate low-frequency content in a blind band-limited numerical dataset, resulting in improved FWI reconstructions [17].

In this study, we investigate the impact of using a 2D CNN architecture to perform low-frequency extrapolation on 2D samples of signal data. The rationale for this approach was from the observation that sequential traces acquired across the elements of transducer arrays provide spatial context to the received data that could complement the time-variation information available in individual traces. For this reason, it may be beneficial for a CNN to be trained to preserve wavefront structure along both the time axis and these consecutive traces. For this task, we used a U-Net architecture, a CNN originally developed for biomedical-image segmentation [21]. It is now widely used as an image-transformation tool, including for image-completion and -sharpening tasks [22,23].

**Figure 1:**
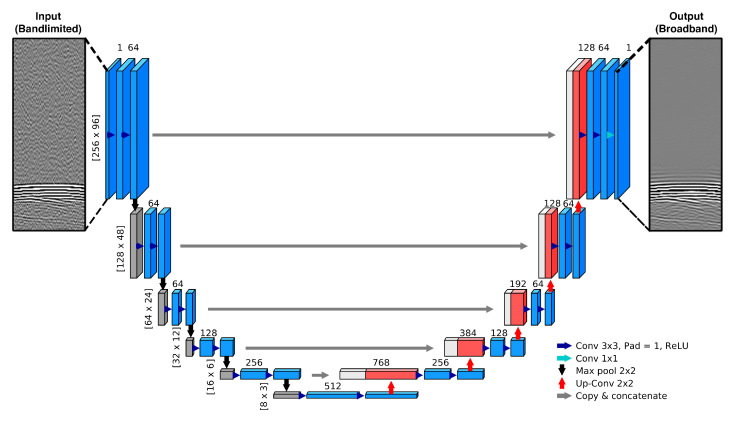
U-net based CNN architecture for extrapolating low-frequency signal content for 2D input samples of ultrasound data. Plotted volumes represent feature maps, with x,y dimensions given at the start of each row, while values above these blocks denote the number of channels. Grey blocks, downsampling steps; red blocks, upsampling steps; white blocks, corresponding feature maps that were copied over from the contracting steps.

The U-Net consists of a contracting path where successive convolutional, ReLU, and max-pooling operations are applied to extract greater amounts of feature information as spatial information is reduced. This is followed by an expansive path where successive upsampling is applied to increase spatial resolution, allowing for the network to produce an output with the same image dimensions as those of the input (Figure 1). A notable feature of a U-Net is the use of concatenated skip connections, whereby feature maps from the contracting path are reused by concatenating them with feature maps of the expansive path. This allows for the network to preserve spatial information when forming the output image that would otherwise be lost. Input images were defined as 256×96 samples of ultrasound data (this corresponds to a window of 256 times samples taken across the 96 elements of a P4-1 probe shot gather for a given source).

### 2.2. Bandwidth-Extension Network Training

Synthetic USCT training data were generated by solving a numerical wave equation to simulate the propagation of acoustic waves through realistic SoS breast models. This allowed for corresponding band-limited and broadband USCT datasets to be generated for a range of different breast imaging problems, providing both the input and target data required to train the bandwidth extension CNNs by supervised learning. To avoid data overfitting, these datasets were created by imaging 8 randomly selected 2D slices from the OA-Breast database models. From the 8 sets of generated data, 4 were selected for training data, 2 were selected for use as validation data during training, and the remaining 2 were selected for testing. The set of transducer-element positions used to generate these datasets was modelled after the experimental acquisition system discussed in Section 2.4.2, which consists of a ring of 16 P4-1 probe positions around the breast models being imaged. Each of the resulting datasets provided 294,912 unique traces that could be randomly sampled for training the 1D CNN model, or 3072 unique sets of 96 element shot gathers for training the 2D CNN model. The augmentation of these data was performed by applying a random shift to the 256 time sampling window for each selected input datum (±144 time steps) and by applying a random flip to the order of the traces being sampled. By applying these augmentation steps, a total of ∼40 million unique 1D traces and 7,077,888 unique 96 trace shot gathers could be sampled for training the low-frequency extrapolation CNN models.

**Figure 2:**
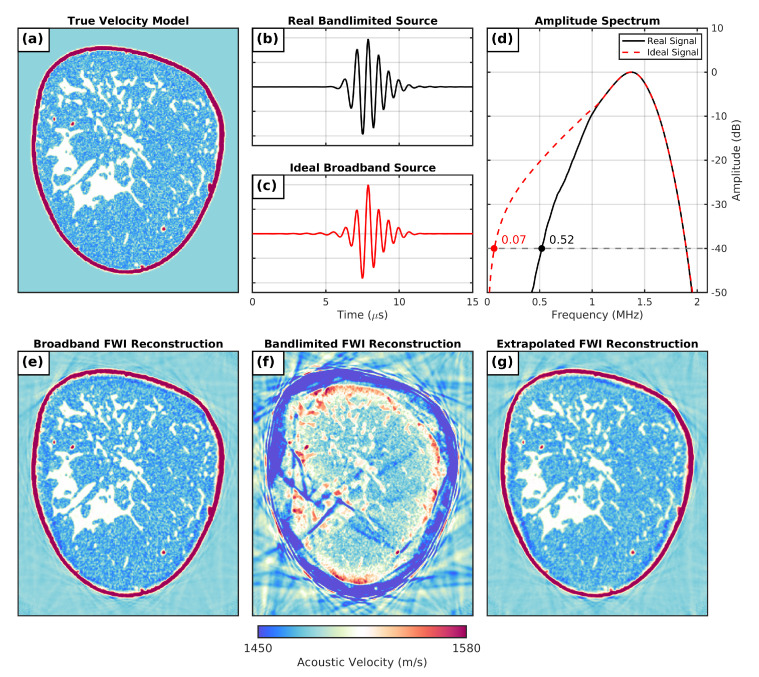
Performance of our 2D U-Net low-frequency extrapolation in FWI. (**a**) Coronal slice of a realistic numerical breast model; (**b**) narrowband source signal extracted from real P4-1 transmission data and (**c**) corresponding ideal broadband source signal; (**d**) amplitude spectrum of real and ideal source signals; (**e**) full-waveform inversion of broadband ultrasound data using ideal source signal; (**f**) full-waveform inversion of bandlimited ultrasound data using real source signal; (**g**) full-waveform inversion of narrow-band ultrasound data after being fed through a low-frequency extrapolation CNN.

To ensure that the synthetic input data realistically represented real-world band-limited data, these datasets were generated using an input signal sampled from transmissions of the P4-1 probes. An example of the recovered P4-1 signal can be seen plotted in Figure 2b, which had a lower frequency limit at −40 dB of 0.52 MHz (as seen in the amplitude-spectrum plot for this signal in Figure 2d). According to the findings of the synthetic FWI study by Calderon Agudo et al. [12], this suggests that the resulting datasets generated with this signal would have insufficient low-frequency content to mitigate cycle-skipping effects when running FWI. To demonstrate this, FWI was run (frequency stepping sequence, 0.50 to 1.20 MHz, 208 iterations) using a synthetic band-limited dataset generated by imaging the SoS breast model seen in Figure 2a using the P4-1 input signal. To perform this inversion without using any prior information about the breast model, a homogeneous water starting model set to 1484 ms${}^{-1}$ was used. As shown in Figure 2f, this inversion was not able to successfully recover the true breast SoS model from this starting model, supporting the assumption that these datasets are not suitable for breast imaging using conventional FWI.

To generate the corresponding broadband target data, each simulated breast-model acquisition was repeated while instead using an ideal broadband source. This signal was designed to be both in-phase and morphologically similar to the observed P4-1 signal, but with greater signal content at lower frequencies. To create this signal, the high-frequency content of the P4-1 signal in the frequency domain (for amplitudes greater than 1.00 MHz) was combined with the lower-frequency signal content of an ideal 3-cycle broadband pulse. The resulting hybrid spectrum of these two signals had greater amplitudes at lower frequencies in Figure 2e than the original P4-1 signal did with a new lower −40 dB frequency limit of 0.07 MHz. An inverse fast Fourier transform of this hybrid frequency spectrum was then used to produce the ideal broadband signal seen plotted in the time domain in Figure 2e. To demonstrate the impact of using USCT data with this additional lower-frequency content, the breast model in Figure 2a was once again imaged, this time while using the ideal broadband source signal or generated broadband testing data. By running FWI with these broadband data (frequency-stepping sequence, 0.20 to 1.20 MHz, 208 iterations), a high-quality reconstruction of the breast model was recovered from a water starting model, as shown in the final iteration of this inversion in Figure 2e.

In addition to using an input signal sampled from real data, realistic noise designed to be similar noise to that observed when imaging with P4-1 probes was also added to the input datasets so that they would closely match real experimental data. This was achieved by training a simple Pix2Pix generative adversarial network (GAN) to produce noise maps until it was unable to distinguish between generated and real noise sampled from P4-1 imaging data. The target broadband data, however, did not receive this additional noise to encourage the network to suppress noise present in the input data, and to attempt to only preserve useful ultrasound signal content. These dataset pairs were then downsampled to reduce the computational load on GPUs.

### 2.3. Bandwidth-Extension Network Evaluation

Once trained, both the 1D and 2D bandwidth extension CNNs were evaluated by applying them to the band-limited input data of 15 additional synthetic breast imaging USCT testing datasets. These were generated using the same method as that described in Section 2.2 while only sampling breast models from OA-Breast data that had not already been used to create training data. For each of these cases, the resulting band-limited input dataset were passed to both CNNs to generate the extrapolated broadband data. For each test case, FWI was then performed across the different USCT datasets, including band-limited input data, ideal broadband data, and two extrapolated broadband datasets generated using the 1D CNN and U-Net-based 2D CNNs. All inversions were run using a homogeneous water starting model with a water velocity value of 1484 ms${}^{-1}$.

As expected, none of the FWI reconstructions using band-limited data (frequency-stepping sequence, 0.50 to 1.20 MHz, iterations = 208) was able to successfully recover the true breast SoS models, and evidence of cycle-skipping artefacts was seen in all cases (as demonstrated by Figure 2f). Conversely, FWI reconstructions performed with ideal broadband datasets (frequency-stepping sequence, 0.20 to 1.20 MHz, iterations = 208) were all found to successfully recover the true numerical breast models for each test case (as demonstrated by Figure 2e). Lastly, FWI reconstructions using all extrapolated broadband datasets were also found to successfully recover the true SoS breast models for each test case. This suggests that both CNN solutions were able to both recover missing low-frequency content in the input data and, after performing this operation, to preserve the high-resolution imaging data of the input signal. An example of one of these successful FWI reconstructions using extrapolated broadband generated with the 2D CNN solution is shown in Figure 2g.

To compare the performance of the 1D CNN and 2D CNN bandwidth-extension models, the RMS error between FWI reconstructions using extrapolated datasets generated using both methods and the true SoS models was computed for each of the 15 test-case slices. The RMS error found for all 1D CNN reconstructions was 5.287 ± 0.448 ms${}^{-1}$, whereas the RMS error for 2D CNN reconstructions was 4.530 ± 0.485 ms${}^{-1}$. These results show that there were significantly smaller RMS values when using the 2D CNN architecture then when using the 1D CNN method, confirming our hypothesis that including spatial information in the network results in more accurate predictions of low-frequency content in USCT data.

**Figure 3:**
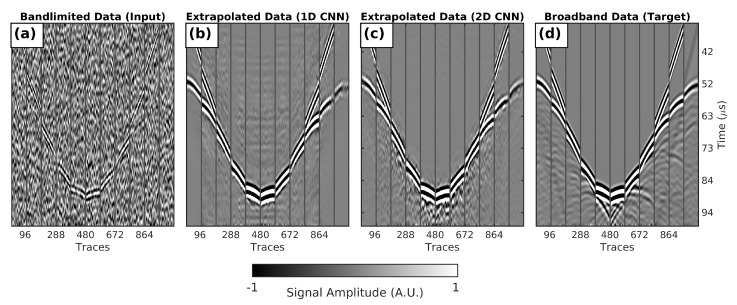
Testing USCT data generated by imaging numerical breast models provided by the OA-Breast database. Each set of 96 traces represents the received signal by the 96 elements of a P4-1 probe at 1 of the positions in the ring array. (**a**) Shot gather simulated using a realistic narrow-band P4-1 source signal and noise; (**b**) shot gather after feeding narrowband signal data into the 1D CNN proposed by Sun et al.; (**c**) shot gather after feeding the narrowband signal data through our U-Net-based 2D CNN; (**d**) shot gather simulated using a ideal broadband source signal without noise.

A closer look at the impact of the low-frequency extrapolation CNN models on ultrasound-signal data is shown in Figure 3, which shows collected data using one of the 15 in silico testing breast models. This includes traces plotted from the input band-limited data, ideal target broadband data, and the extrapolated broadband data generated by the CNNs. Each block of 96 traces represents the 96 receiving elements for probes moving clockwise in the ring array. To highlight the effect of the extrapolating low frequencies, a 0.10–0.25 MHz band-pass filter was applied to all datasets. Transmitted and reflected ultrasound signals were clearly present in the original broadband panel (Figure 3d), but this information was mostly lost in the band-limited data (Figure 3a). As shown in Figure 3b,c, this missing low-frequency signal was successfully recovered after applying the CNNs. However, in the 1D CNN traces, there were notable noisy artefacts that were not present in the true broadband traces or 2D CNN output traces. Again, this is evidence that it is beneficial to include spatial information during low-frequency extrapolation, as the network learnt that, while the noise did not have good lateral continuity, the USCT signal did.

**Figure 4:**
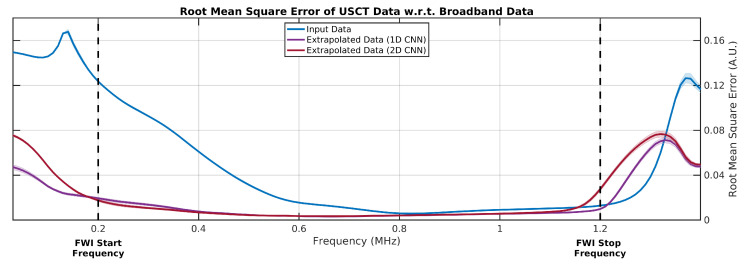
Root-mean-square (RMS) error of synthetic breast USCT data with respect to ideal broadband data with frequency. Error measurements calculated at different frequency bands by applying an FIR band-pass filter to sample USCT signal data within a 50 kHz wide sliding window centred at frequencies in the range of 0.03–1.50 MHz. Trend lines represent mean RMS error observed for USCT data generated for all 15 testing breast models; shaded area at each trend line represents observed error within these measurements. FWI starting and stopping frequency limits were assigned to be 0.20 and 1.20 MHz, respectively, from observing that extrapolated data tended to closely match the ideal broadband data in this frequency range, as indicated by the low error values.

Despite the successfully reconstruction of SoS breast models when using conventional FWI for all generated extrapolated datasets, there were notable limits as to how closely the CNNs were able to transform the input USCT signal into the corresponding ideal broadband target signal. This can be observed by calculating the RMS error of each USCT dataset with respect to the ideal broadband case for different frequency bands, as seen plotted in Figure 4. As expected, the RMS error of the input USCT data for frequencies below the −40 dB data lower-frequency limit (0.52 MHz, as shown in Figure 2d) was significantly lower for the extrapolated data. This supports the observation that these extrapolated datasets match more closely with their ideal broadband cases compared to the input data. However, despite the ideal broadband having a −40 dB lower frequency limit of 0.07 MHz (as seen in Figure 2d) this error can be seen to increase for both extrapolated data cases for frequencies less than 0.2 MHz. This is due to the recovered signal content becoming gradually less coherent with the true broadband low frequency signal for frequencies tending towards 0 Hz. Likewise, an upper limit of frequencies with low RMS error values is visible around 1.20 MHz; after that, the RMS error increased when moving towards higher frequencies. The extrapolated and ideal broadband datasets appeared to most closely match within the 0.2–1.2 MHz frequency range for all 15 testing breast model cases. For this reason, a starting frequency of 0.20 MHz and a stopping frequency of 1.20 MHz were chosen when running FWI with both the ideal broadband and the extrapolated broadband datasets.

### 2.4. Ultrasound-Tomography Acquisition

**Figure 5:**
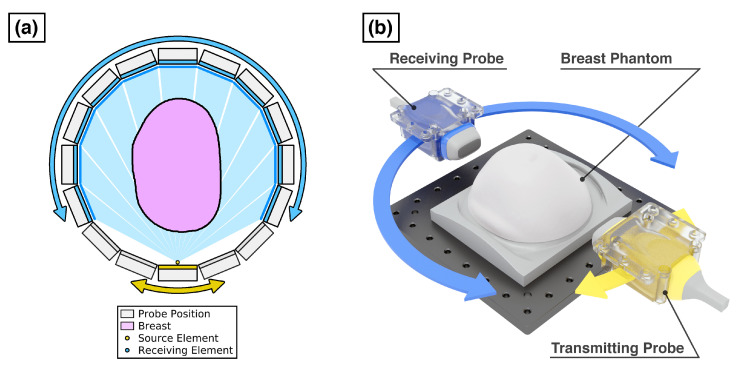
Dual-probe ultrasound tomography acquisition system. (**a**) Top-side view of acquisition ring formed by rotating receiving probe (blue) to acquire the signal at 11 opposing probe positions for all 16 source probe positions (yellow). Direct ray paths between a given source element shot to all receiving elements plotted in light blue; (**b**) 3D rendering of transmitting and receiving ultrasound probes relative to breast phantom.

Two ultrasound imaging systems where used to acquire the experimental data. These were the Dual-Probe USCT Acquisition system developed at Imperial College London by the Transmission and Reflection Ultrasound Tomography (TRUST) research group, which was used to acquire USCT data for running FWI; and the Multi-Modal Ultrasound Breast Imaging platform (MUBI) developed at the Spanish National Research Council [24], which was used to obtain reflectivity images. Both systems used pairs of independently rotated cardiac probes that could be positioned to allow for ultrasound data to be acquired for different source and receiver configurations. A 3D rendering of how a receiving probe (shown in blue) could be positioned relative to a transmitting probe (shown in yellow) while imaging the CIRS breast phantom is shown in Figure 5b. These acquisitions were performed in water tanks to provide a transmission medium between probes and breast phantom. The full volume of the phantom was acquired by adjusting its position in the vertical direction so that it could be imaged over several slices.

#### 2.4.1. Dual-Probe USCT Acquisition System

USCT was acquired using a system using dual 96-element P4-1 cardiac probes and a 256 channel Vantage ultrasound-imaging system (Verasonics, WA, USA). Motors to control the rotation of these probes were a large aperture rotary motor (Standa, Vilnius, Lithuania) and a PRMTZ8/M motor (Thorlabs Inc.,Newton, NJ, US). To acquire the full USCT datasets for 2D slices of the breast phantom in both reflection and transmission, an imaging sequence was designed to simulate the data that could be acquired with a fixed ring array of ultrasound transducers. This resulting dataset then consisted of the acquired ultrasound signal for all possible source–receiver transducer element pairs. As illustrated in Figure 5a, this was achieved by rotating these probes to 16 possible source and receiver positions around the breast phantom to form a ring ∼200 mm in diameter. When the transmitting probe was placed at any one of these positions, the receiving prove could then be rotated to acquire data over the 11 opposing receiving positions (as indicated by the blue probe arrays for the single yellow transmitting element shown in Figure 5a). The full sequence consisted of repeating this acquisition for all 16 transmitting positions of the ring array.

Due to the nature of the scanning method used in this system, both the relative transducer positions and transmitted source wavelets were initially considered to be unknown. These values were, therefore, estimated by extracting TOF information from a calibration USCT dataset acquired prior to placing the CIRS phantom for imaging. As the width of a P4-1 transducer element (width = 2.45 × 10${}^{-1}$ mm) was less than the minimal wavelength found in the observed data ($\lambda_{min}$ = 9.25 × 10${}^{-1}$ mm), these elements were considered to be point sources and receivers in a 2D imaging plane. Element localisation was, therefore, performed by minimising the misfit between the computed travel of these modelled element points and the travel times experimentally found for all source–receiver element pairs. This was achieved by posing the problem as a nonlinear least-squares optimisation that could be iteratively solved using a Gauss–Newton algorithm [25]. These optimised transducer positions could then be used to provide a wavelet estimate for each source. This was achieved by first applying a normal move-out (NMO) correction [26] to all received signals for a given source, and then taking the mean signal across the resulting stack of coherently aligned traces. An example of a recovered source wavelet from these P4-1 probes is shown in Figure 2b.

#### 2.4.2. Reflectivity-Acquisition System

Reflectivity images were acquired with the Multi-Modal Ultrasound Breast Imaging (MUBI) platform developed by CSIC [24]. A single probe of 3.20 MHz, 128 elements, and 0.22 mm pitch (Prosonic, South Korea) was rotated around the phantom to the 16 positions of the probe ring array used by the Imperial College USCT system. At each probe position, a sector-scan reflectivity image was obtained, with 256 scan lines equally spaced between −60 and 60°, emission focus at 100 mm depth, and dynamic depth focusing on reception. The resulting images were formed in real time by a SITAU-112 ultrasound system with 128 parallel channels (Dasel SL, Madrid, Spain).

These 16 images were combined using the full-angle spatial-compound technique [20]. For each sector image, the interface between water and phantom was automatically detected from ultrasound data, and the propagation direction of each scan line inside the phantom was corrected to account for the beam refraction. This process was repeated by changing the assumed average SoS of the tissue (unknown) between 1400 and 1600 ms${}^{-1}$ with an optimization process that uses image sharpness as a beamforming quality measure to find the optimal SoS value [27]. Then, the 16 refraction-corrected images were converted into a common rectangular grid by bilinear interpolation and accumulated to obtain the full-angle spatially compounded image.

## 3. Results

In this section, we present results from applying our FWI algorithm and 2D CNN low-frequency extrapolation solution to two breast imaging problems with band-limited USCT data: (1) a 2D in silico dataset where the ground-truth breast phantom model was unknown, and (2) experimentally acquired 2D data by imaging a realistic CIRS breast phantom using the dual-probe acquisition system. Extrapolated broadband data were approximated for each problem set by feeding band-limited input data into our trained network, allowing for us to study the impact of extrapolating low-frequency signal content on the FWI reconstructions. To validate these results, the CIRS acoustic breast phantom was also imaged using reflection ultrasound tomography and X-ray computed tomography (CT).

### 3.1. In Silico Breast Phantom Experiment

An in silico breast phantom was used to provide both band-limited and broadband USCT data to test our 2D CNN bandwidth-extension solution. This was provided by the MUST 2019 Data Challenge and consisted of an SoS breast model that had been imaged using a ring array of 248 transducers acting as both sources and receivers. FWI parameters, including true source signals, transducer positions, and water SoS were provided alongside this USCT challenge dataset. However, given that the aim of this challenge was to achieve the most accurate reconstruction of the numerical breast model, the true used SoS breast model was not released. This, therefore, provided an ideal blind imaging problem for this study.

**Figure 6:**
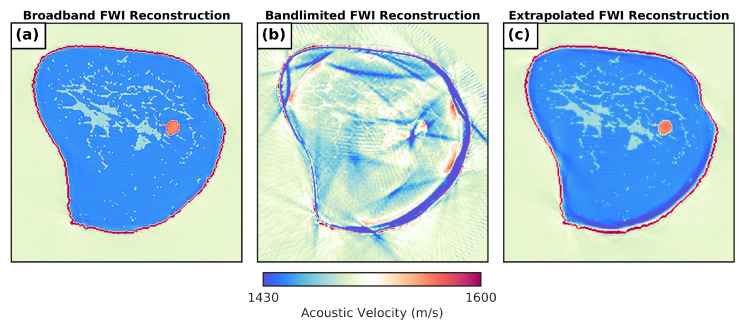
Inversion results for in silico breast imaging data provided by MUST 2019 Data Challenge. (**a**) Full-waveform inversion result using broadband signal data provided for the challenge; (**b**) full-waveform inversion result using narrow-band data acquired by high-pass filtering original broadband dataset; (**c**) full-waveform inversion result using extrapolated broadband data generated using a U-Net CNN to recover missing low-frequency content removed from narrow-band data.

The initial USCT dataset was found to be suitably broadband to overcome cycle skipping (0.20–4.00 MHz above −40 dB), allowing for a successful reconstruction of the numerical breast phantom to be recovered using FWI (frequency-stepping sequence, 0.20 to 1.20 MHz, 208 iterations), as shown in Figure 6a. This dataset was, therefore, used as a broadband reference. To provide a more realistic band-limited dataset, a high-pass filter (FIR, order = 40, cutoff = 0.55 MHz) was applied to the broadband dataset to remove frequency content below 0.50 MHz (this was to reflect the observed bandwidth when imaging with the P4-1 cardiac probes). As shown in Figure 6b, the FWI reconstruction using this filtered dataset resulted in cycle-skipping artefacts (frequency-stepping sequence, 0.50 to 1.20 MHz, 208 iterations). This prevented breast-tissue structures from being correctly recovered. A root mean square (RMS) error of 36.593 ms${}^{-1}$ was found between these band-limited and broadband (ground-truth) FWI reconstructions.

The 2D CNN bandwidth extension solution was then applied to produce an extrapolated broadband dataset. Rerunning the same FWI sequence used for the broadband ground-truth data resulted in an accurate reconstruction of the breast model that was comparable to the original broadband result (Figure 6c). High-resolution breast-tissue structures were also visible in the broadband ground-truth reconstruction, and a significantly reduced RMS error of 6.965 ms${}^{-1}$ was found between the extrapolated and ground-truth results, suggesting that the extrapolated dataset had sufficient low-frequency content to overcome cycle skipping. Inconsistencies in breast-tissue SoS values could be observed in some regions, but most notably along the lower boundary of the phantom. These may have been due discrepancies between how different output traces were encoded with information pertaining to the same model features within the extrapolated low-frequency signal.

### 3.2. CIRS Breast Phantom Experiment

Experimental USCT data were acquired by imaging the CIRS 073 breast phantom that had been designed to realistically reproduce breast morphology and acoustic SoS. This was performed using the dual-probe USCT acquisition illustrated in Figure 5 and discussed in Section 2.4 to image the phantom over 25 coronal slices, each separated by a step size of 2.00 mm.

**Figure 7:**
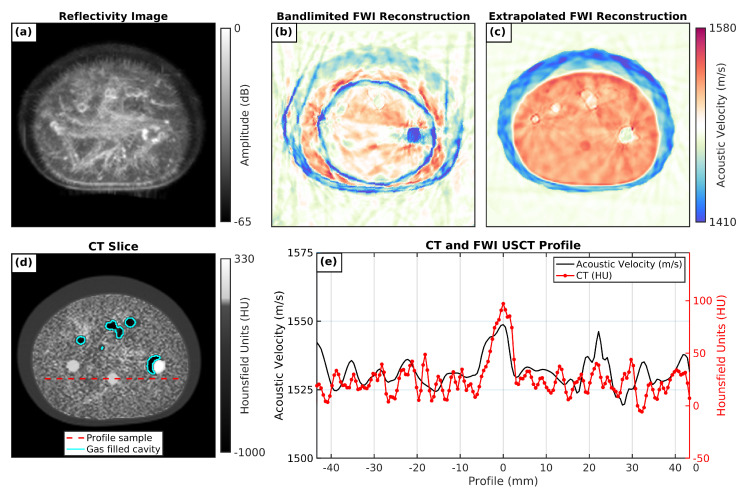
Results from imaging a cross-sectional slice of the CIRS breast phantom. (**a**) Reflectivity image with full-angle spatial-compound technique with refraction correction and 65 dB dynamic range; (**b**) full-waveform inversion reconstruction using using narrow-band data; (**c**) full-waveform inversion reconstruction using using low-frequency extrapolated data; (**d**) CT with gas-filled cavities highlighted in cyan; (**e**) profile of CT and FWI USCT along the phantom in a region with a uniform background and a central area with higher density.

FWI reconstructions of the CIRS breast phantom using USCT data acquired by the P4-1 probes did not appear to have sufficient low-frequency content to overcome cycle skipping. This resulted in numerous imaging artefacts and poorly reconstructed breast structures, as shown in Figure 7b. These results were similar to those of the unsuccessful reconstructions discussed in Section 2.1, where cycle skipping was also observed when inverting the synthetic band-limited USCT data Figure 2f. When running FWI using the extrapolated data, however, the resulting reconstructions provided realistic breast phantom structures that were in good agreement with the micro-CT ground-truth model of the CIRS phantom, as shown in Figure 7d. In both extrapolated FWI and CT images, the phantom consisted of two distinct layers of breast-tissue mimicking material with contrasting density and SoS values to replicate the glandular and subcutaneous fat tissue layers of breasts in vivo. Furthermore, several small closely matching features were seen in both results including scatterers, cavities, and regions of higher density and SoS. However, the small cavities in the CT scan (outlined in cyan in Figure 7d) appeared to contain attenuation values similar to those of the air surrounding the phantom. This suggests that the phantom contained gas-filled cavities that we would not expect to see in real breast tissue. This appears to be a manufacturing defect in the CIRS 073 breast phantom, which was also reported in other publications [28]. Due to the limitations of the numerical-wave-equation solver in the presence of the large contrast between the acoustic properties of air (SoS ≈ 343 ms${}^{-1}$, ρ = 1204 kg $m^{3}$) and breast tissue (SoS ≈ 1400–1700 ms${}^{-1}$, ρ = 900–1057 kg $m^{3}$), the presence of these gas cavities had a detrimental effect on the quality of FWI reconstructions, resulting in reconstruction artefacts at positions that corresponded to these gas cavities.

### 3.3. Reflection Tomography

Figure 7a shows the reflectivity image for a slice of the CIRS phantom. The outer low-velocity layer present in the FWI image and the CT was also observed in the reflectivity image. In this case, it was a hypoechoic region of similar thickness, probably generated by less scattering material used for mimicking fat tissue. In the interior of the phantom, quite a different texture was observed. Filament-like structures were present in the reflectivity images, which could be explained by the nonuniform concentration of scatterers that locally affect material reflectivity but do not significantly change the SoS, at least at the resolution range of the FWI reconstruction. This pattern was also observed in the CT by restricting the greyscale palette for observing subtle density changes (see Figure 7d), which confirmed its presence and discarded a possible reflectivity reconstruction artefact. On the other hand, not all cysts and masses seen in the FWI reconstruction (see Figure 7c) were seen in the reflectivity image. This was expected because some of the simulated lesions had different stiffness than, but similar reflectivity to, those of the background material. This observation reinforces the hypothesis that multimodal ultrasound imaging, combining reflectivity and SoS modalities, provides complementary information that could improve cancer diagnosis.

### 3.4. Quantitative-Analysis Results

In order to evaluate the accuracy of the reconstructed images with the proposed method, quantitative analysis was performed. First, the reconstructed FWI USCT images of the CIRS-073 breast phantom were coregistered with the CT acquisition by using a set of landmarks that could be easily identified in both image modalities, as is shown in Figure 7. Then, three different sets of regions of interest (ROI) were identified that were visible in both the CT and FWI images. These corresponded to a background with similar density to that of soft tissue (CT number between 0 and 50), regions with slightly higher density (CT number between 50 and 100), and ROIs with the highest density (CT number higher than 100). As the identified gas-filled cavities in the CT images were not realistic, they were omitted from quantitative analysis.

**Figure 8:**
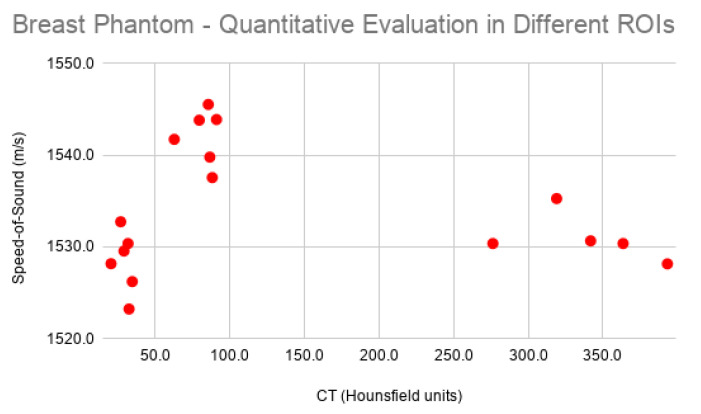
Values of speed of sound of different obtained regions from the FWI USCT images, and their corresponding Hounsfield units from CT.

The analytical results of the values of these regions are summarized in Figure 8. SoS values were significantly higher for regions in Set 2 (moderate high density), while Set 3 (high density) had similar SoS to that of the background (set #1). This agreed with the available information from the CIRS-073 phantom brochure [19]. A profile along the CT and FWI USCT images (Figure 7e) confirmed that there was good agreement between the results of the two independent reconstructions. The region with HU around 100 had a significantly higher SoS (around 1550 ms${}^{-1}$).

## 4. Discussion

FWI could transform ultrasound breast imaging. It can produce images at a comparable resolution to that in mammography but while using a safe, painfree, and more universally applicable solution. However, practical implementations require frequencies below 1.00 MHz to avoid cycle skipping and to reduce computational costs. The lack of sub-megahertz energy with sufficient SNR is common in USCT acquisition devices, as they are often designed to generate data for ray-based imaging methods that require high frequencies to produce images with adequate resolution to be diagnostically useful.

To circumvent the deficit of low-frequency data, we implemented a solution based on a U-Net-based 2D CNN that could successfully extend the bandwidth of ultrasound datasets towards the low end of the amplitude spectrum. The output datasets from the network contained an accurate estimation of what this low-frequency information would be if broader-band transducers were available, and they could produce accurate FWI reconstructions where the original data with missing low-frequency information failed. We demonstrated that it is possible to train our network with in silico datasets, and that the trained network performed well when exposed to data acquired in the laboratory. This suggests that the method is robust against noise present in real data, and that it could learn the mapping between high and low frequencies purely from numerical simulations of ultrasound wave propagation.

The validation on laboratory data resulted in SoS images that were morphologically consistent with X-ray CT and ultrasound reflection tomography, with added quantitative information of physical properties. Nonetheless, our reconstructed images contained artefacts due to the geometry of the acquisition system: transducers were distributed in a ring that moved along its normal axis. Despite the relatively focused illumination on the ring plane, there was significant energy that propagated outside this plane that interacted with the acoustic heterogeneities that existed there. These interactions wre inevitably mapped onto the data, and the final images thereby suffered from a lack of resolution along the axis perpendicular to the ring plane. This problem could be solved by simply using data acquired with a 3D system that could capture ultrasound energy at all angles. The extension of our method to 3D datasets is straightforward. Comparisons of the performance of 1D and 2D CNNs on our 2D datasets showed some differences between the outputs of the two networks, but most of the performance gains that we observed in the 2D case could have been due to the presence of the spatial-distribution context. Extending the network inputs from 2D to 3D did not benefit as much from this extra information because the third added dimension contained already captured information in the addition of the second dimension. In other words, the difference between 1D and 2D was that our data transformed from a pure time series of values into a collection of spatially ordered time series; the third dimension only added a relatively redundant spatial dimension.

The continuous increase in computational capabilities is creating a shift in ultrasound imaging. Wave-equation-based reconstruction methods such as FWI continue to reconstruct images with better resolution and accuracy than those of ray-based alternatives. However, there is still a large computational cost to jump between the two that limits the applicability of wave-based methods to lower frequencies than those used in ray-tracing methods. Our proposed method offers a solution that allows for both ray-tracing and wave-based ultrasound imaging on the same data by extending the original bandwidth.

Lastly, the proposed method can not only be used for improving FWI. A reliable method to extend the frequency content of band-limited ultrasound signals can also be used to overcome the physical limitations of PZT crystals, extending the use of existing US hardware for other frequency ranges or predicting produced data by an ideal US hardware system.

## 5. Conclusions

In this work, we showed how a 2D CNN such as U-Net can be used to extend the frequency content of USCT data for FWI breast imaging. Realistic in silico USCT data were generated to provide input and target data to train a CNN to perform this bandwidth-extension operation. The performance of the CNN was then evaluated using simulated and experimental FWI breast phantom imaging problems with band-limited input data. We demonstrated how applying the CNN as a prepossessing step to the input data could be used to recover missing low frequencies, allowing for FWI to be run without cycle skipping. By doing so, we were able to recover high-resolution quantitative SoS models of the breast phantoms despite starting with input data that were not suitable for FWI breast imaging. The preprocessing of acquired USCT data with trained bandwidth-extension CNN solutions may become a standard procedure prior to running FWI, similar to the application of some currently used filters. Lastly, this approach could be used to overcome the physical limitations of ultrasound hardware that images with suboptimal bandwidths.

## Data Availability

The true acoustic breast models used to generate the training and validation data for this study were sampled from the OA-Breast Database created by the Computational Imaging Science Laboratory at Washington University in St. Louis [18]. This data was made available under the Open Database License and can be found at https://anastasiolab.wustl.edu/downloadable-content/oa-breast-database/, (accessed on 30 June 2021). The blind synthetic breast imaging problem discussed in Section 3.1 was created for the 2nd International Workshop on Medical Ultrasound Tomography USCT Data Challenge (2019). This data is avaliable at https://usct.gitlab.io/datachallenge2019/data/, (accessed on 30 June 2021).
